# A Mini-Review on Electrocatalytic Self-Cleaning Membrane Materials for Sustainable Fouling Control

**DOI:** 10.3390/membranes15070191

**Published:** 2025-06-25

**Authors:** Honghuan Yin, Zhonglong Yin

**Affiliations:** 1 Tianjin Key Laboratory of Refrigeration Technology, Tianjin University of Commerce, No. 409 Guangrong Road, Beichen District, Tianjin 300134, China; 2 Jiangsu Key Laboratory of New Power Batteries, Jiangsu Collaborative Innovation Center of Biomedical Functional Materials, School of Chemistry and Materials Science, Nanjing Normal University, Nanjing 210023, China

**Keywords:** electrocatalytic membrane, self-cleaning, membrane fouling, microfiltration, ultrafiltration, nanofiltration

## Abstract

Although membrane technology has been widely applied in water treatment, membrane fouling is an inevitable issue that has largely limited its application. Benefiting from the advantages of green power, easy integration and low chemical consumption, electrocatalytic membrane (ECM) technology received attention, using it to enable electrically driven self-cleaning performance recently, making it highly desirable for sustainable fouling control. In this work, we comprehensively summarized the conventional (e.g., carbonaceous materials, metal and metal oxide) and emerging (e.g., metal–organic framework and MXene) materials for the fabrication of an ECM. Then the fabrication methods and operating modes of an ECM were emphasized. Afterwards, the application of different ECM materials in membrane fouling control was highlighted and the corresponding mechanism was revealed. Based on existing research findings, we proposed the challenges and future prospects of ECM materials for practical application. This study provides enlightening knowledge into the development of ECM materials for sustainable fouling control.

## 1. Introduction

Membrane technology played an irreplaceable role in wastewater treatment and resource recovery owing to its advantages of being environmental friendly, showing high pollutant removal and having a small footprint [1]. However, foulants in wastewater accumulated on the membrane surface and caused membrane fouling, which markedly reduced the permeability and the lifetime of the membrane and this was regarded as the “Achilles heel” of membrane technology [2]. Inorganic, organic, colloidal and microbial foulants caused inorganic, organic, colloidal and biological fouling. Combined fouling (e.g., organic–inorganic fouling, organic–biological fouling) may also appear when different kinds of foulants coexist in wastewater, which usually induces more serious fouling than individual fouling [3,4].

**Figure 1:**
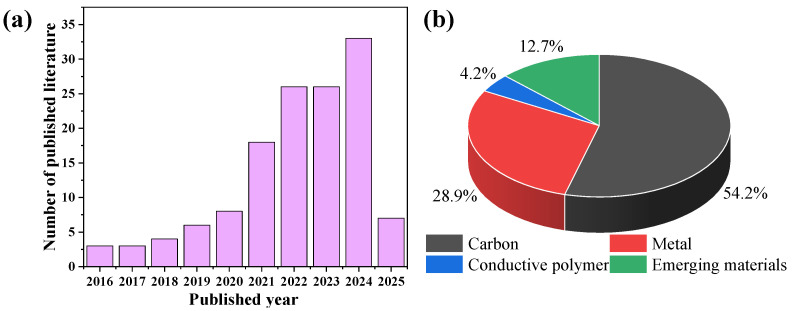
Recent publications related to ECM technology for water treatment within 10 years (**a**) and summary of publications on ECM materials for water treatment (**b**). The data are taken from http://apps.webofknowledge.com (accessed on 18 May 2025).

Currently, to mitigate membrane fouling, long-process pre-treatment (e.g., coagulation, adsorption, oxidation) is inevitably used in removing the foulants in industry [5,6]; moreover, chemical cleaning using cleaning agents (e.g., acid, base and oxidants) is usually employed to recover membrane performance when irreversible fouling occurs [7], facing the challenges of a high chemical input, operating cost, carbon emissions and secondary pollution [2]. Therefore, it is appealing to develop more sustainable and scalable membrane fouling control strategies. Recently, the catalytic membrane has attracted intensive attention in membrane fouling control, which combines the dual-functional advantages of membrane and catalysis technology [8]. The foulants on a membrane can be mineralized or degraded into smaller degradation products with lower fouling propensity with the membrane [9]. Among recent technology, electrocatalytic membrane (ECM) technology presented unique merits of green power (e.g., wind and solar), easy integration and no addition of expensive and hazardous chemicals, making it a promising candidate to enable efficient fouling mitigation via the direct oxidation (electron transfer-mediated) or indirect oxidation (active species-mediated) pathway [10,11]. We searched the published literature from 2016 to 2025 from the Web of Science using “electrocatalysis”, “fouling” and “membrane” as the keywords. The published literature numbers sharply increased in the last 5 years (Figure 1a).

The rational design of ECM materials with high catalytic activity, stability, low-cost and scalable fabrication is the key step to achieve the efficient degradation of pollutants for sustainable membrane fouling control under low energy consumption. ECM materials mainly included carbon (e.g., graphene oxide (GO), carbon nanotube (CNT)), metal and metal oxide (e.g., Magneli phase Ti_4_O_7_, SnO_2_), and a conductive polymer (e.g., polyaniline (PANI), polypyrrole (PPY)) [12,13], which accounted for 54.2%, 28.9% and 4.2% according to the published literature in the past ten years (Figure 1b), respectively. The combination of different materials is promising to inherit the merits of each material while overcoming its shortcomings, such as FeOCl@CNTs [14], and reduced graphene oxide-zinc oxide [15]. In addition, with the development of material science, emerging ECM materials were reported with unique structures with a percentage of 12.7%, such as metal–organic frameworks (MOFs) [16], covalent–organic frameworks (COFs) [17] and MXene [18,19]. Although their proportion is lower than conventional ECM materials, they are promising to play a greater role in the future owing to their exceptional porosity, designability and catalytic activity. The merits and drawbacks of common ECM materials were provided in Table 1. However, the scalable fabrication of an ECM with a low cost, high stability and electrocatalytic activity is still challenging.

**Table 1:** The merits and drawbacks of common ECM materials.

ECM	Merits	Drawbacks
CNTs	High electrical conductivity [20]; Tunable surface structure [20]; Larger specific surface area; Good chemical stability and mechanical properties.	Poor electrocatalytic activity [20]; Low overpotential for the competitive oxygen evolution reaction [12]; High cost.
Transition metal oxides	Tunable electronic structure [21]; Environmentally friendly; Efficient generation of ROS [16].	Easy passivation [12]; High preparation costs [22]; Low intrinsic catalytic activity;
PANI	Low cost [23]; Good redox ability [23]; High chemical stability [23].	Generation of organic waste during synthesis process [24]. Limited conductivity; Poor selectivity for desired pollutants;
MXene	Large number of active sites [25]; Adjustable surface chemical properties [25]; Fast charge and electron transfer ability [25].	Easy agglomeration [25]; Poor processibility [25]; Poor oxidation resistance [26].
MOFs	Highly adjustable pore architectures [27]; Plentiful supply of active sites [27]; Rich microenvironment inherent [27]; Larger specific surface area [27].	Poor conductivity [28]; Low stability [28]; Easy agglomeration [28]; Risk of metal leaching [29]; Lack of exposed active sites [29].
COFs	Adjustable structure [30]; Good chemical stability [30]; Larger specific surface area [30]; Tunable conductivity [30].	Inherently low electrical conductivity [31]; Limited accessibility of active sites [31]; Difficulty in synthesis.

**Figure 2:**
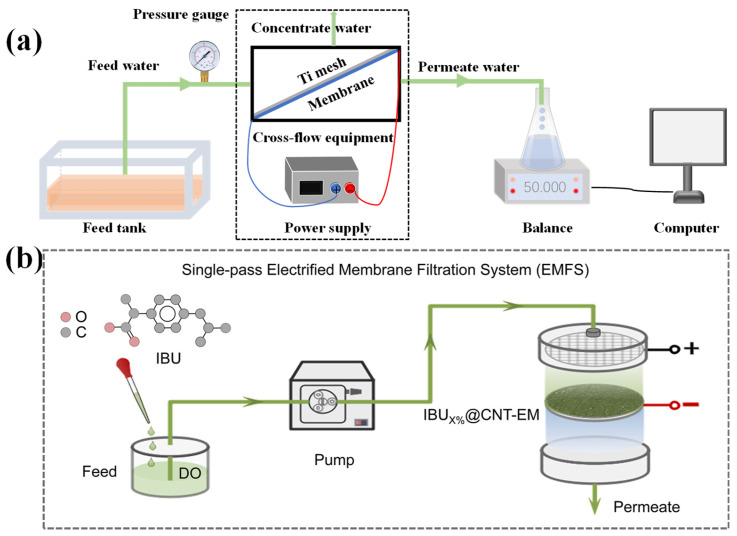
Schematic diagram of the operating modes of an ECM. (**a**) Cross-flow membrane filtration system [9]; (**b**) flow-through membrane filtration system [36].

ECMs can be classified as anode ECMs and cathode ECMs for the degradation of organic pollutants and disinfection in direct and indirect oxidation mechanisms, which holds great potential for water remediation and reclamation [13]. The operation modes of ECMs can be categorized into two distinct types: flow-by (Figure 2a) and flow-through (Figure 2b) filtration systems [32]. The application of ECM technology can also extend to drinking water for the removal of emerging organic contaminants (e.g., antibiotics, per- and polyfluoroalkyl substances (PFASs), endocrine disrupting chemicals (EDCs) and persistent organic pollutants (POPs)) that are difficult to remove with conventional wastewater treatment methods [12]. However, many works have focused on the removal of specific pollutants (especially for the small molecules) for wastewater treatment, while membrane fouling control was not considered. Generally, organics with a high molecular weight (e.g., biopolymers, soluble microbial products (SMP)) were regarded as the main foulants causing severe membrane fouling [33,34], while their structure was quite different from the small molecular pollutants in wastewater. Therefore, an ECM with high electrocatalytic activity toward small molecular pollutants does not always mean that it has good membrane fouling control ability. Moreover, the fouling control mechanism is rather complicated owing to the complex water matrices [2]. In view of the exceptional performance of an ECM in sustainable fouling control and the huge potential for implementation, it is timely for a review. Recently, several pioneering reviews drew conclusions about the recent advances of electrocatalytic membranes in wastewater treatment [8,10,35], while rare reviews systematically summarized and commentated on the fouling control performance and mechanism of electrocatalytic membranes.

This mini-review aims to summarize findings on ECM materials for membrane fouling control during water treatment, developing fabrication methods and ECM materials being designed for suitable for environmental and industrial applications. In addition, we provided a brief overview of fouling control performance and the mechanisms of anode and cathode ECMs in different application scenarios. Then the challenges and future trends of ECMs for sustainable fouling control were proposed. Overall, we believe that this review will provide guidance for the development of ECM materials in water treatment.

## 2. Electrocatalytic Membrane Materials

### 2.1. Carbonaceous Materials

Carbonaceous materials are mainly composed of sp^2^ carbon hybridized with delocalized π–π electrons, which present high electrical conductivity and porosity [37], including zero-dimensional carbon quantum dots, one-dimensional CNTs, two-dimensional graphene analogs, three-dimensional boron-doped diamonds (BDDs), and carbon fibers. Also, they are easy to modify by doping metal or heteroatoms, therefore they are intensively investigated as ECMs. However, their oxidation ability was insufficient because of their intrinsic nature of an “active” electrode that interacts with electrogenerated reactive oxygen species (ROS) [12]. Moreover, the oxygen evolution potential (OEP) of carbonaceous materials is normally lower than 0.4 V, and they are prone to exhibit a competitive oxygen evolution reaction (OER) [37].

Among them, graphene was firstly fabricated through micromechanical exfoliation [38] and then researchers made great advances in its membrane separation and electrochemical application owing to its tunable interlayer channel, facile and large-scale production and high electrical conductivity [39]. Graphene oxide (GO) is composed of layers of GO and contains oxygen functional groups, while reduced graphene oxide (rGO) has more sp^2^ hybridization, and higher electrical conductivity and hydrophobicity than GO [40]. It is usually combined with metal to enhance electrocatalytic activity (e.g., rGO/Ti_4_O_7_/SnO_2_-Sb [41]), where the oxygen functional groups can provide the binding sites with the metal. rGO/ZnO was also reported to enhance the electron transfer rate for superior •OH generation, which enabled 90% flux recovery after dye fouling [15]. Although metal can act as active sites for enhanced catalytic activity toward pollutant degradation, the possible leaching of metal may cause the deteriorated performance of the ECM and the pollution of the permeate. Therefore, it is imperative to prevent the agglomeration of composites and firmly fix the metal on GO. In addition, doping heteroatoms (e.g., N, S, P, B) into graphene analogs is a powerful strategy to fabricate metal-free ECMs, which exhibit less π–π stacking, optimize the electron structure, introduce defects and change the microenvironment of active sites, therefore drawing intensive attention recently [42]. For example, N doping into graphene markedly improved the cathodic production of H_2_O_2_ for the degradation of pollutants, which was influenced by the nitrogen species. Pyrrole-N and graphite-N enhanced the H_2_O_2_ selectivity since they are favorable to produce oxygen-containing substances (e.g., *OOH) [43]. Pyridinic N-rich graphitic carbon nitride-loaded GO was also reported with a high molecular oxygen activation ability for the electro-generation of H_2_O_2_; moreover, oxygen-containing groups on GO facilitated an 2e^−^ oxygen reduction reaction (ORR) and pyridinic N boosted the decomposition of H_2_O_2_ into •O_2_^−^ [44]. B doping imparts graphene with a lower negative charge, which is beneficial for the adsorption and catalysis of positively charged pollutants [45]. Notably, the excessive doping of heteroatoms on a graphene ECM may reduce the stability and cause the occurrence of a competitive water splitting reaction. Therefore, the moderate doping and precise control of the heteroatom species and heteroatoms–graphene interaction are important to optimize ECM performance.

**Figure 3:**
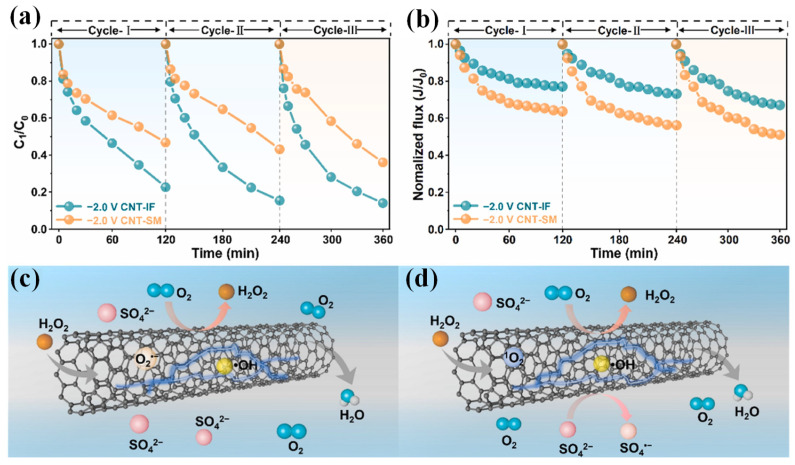
(**a**) Degradation curve and (**b**) normalized flux of humic acid in three cycle experiments at −2.0 V for CNT internal fabrication (CNT-IF) and CNT surface modification (CNT-SM) membranes; Degradation mechanism for (**c**) CNT-IF and (**d**) CNT-SM systems [48]. C_1_/C_0_ represented the ratio of the residual concentration of pollutants to the initial concentration of pollutants.

CNTs consist of “rolled-up” graphite sheets, including single-walled carbon nanotubes (SWCNTs) and multiwalled carbon nanotubes (MWCNTs), which are additional important carbonaceous materials in electrochemical applications owing to their high electrical conductivity, mechanical stability and porosity [46]. CNT-based ECMs can degrade pollutants through direct and indirect oxidation (•OH) pathways. Yang et al. [36] proposed that the augmented adsorption of ibuprofen on CNT cathode ECMs lowered H_2_O_2_ formation owing to the obstruction of electron transport, while the proper adsorption of ibuprofen boosted H_2_O_2_ production since the moderate hydrophilic–hydrophobic structure facilitated the accessibility of O_2_ and H^+^ to active sites and hence enhanced the reaction activity. Consequently, membrane fouling by organic foulants and microorganisms was mitigated. This study suggests that the adsorption of pollutants in wastewater on catalysts may change their structure and physicochemical properties, which influenced the activity and stability. It is important to unveil the different kinds of pollutants and water matrices that affect ECM properties, which helps to optimize the antifouling and self-cleaning performance in order to face the challenges of complex water matrices. The orientation of the CNTs’ layers influenced the electrocatalytic degradation of a UF-based ECM with high permeability [47]. In addition, the continuous CNT network in the PVDF membrane matrix reduced the electron transfer resistance, therefore facilitating ROS generation and alleviating the membrane fouling caused by humic acid [48]. This study evaluated the performance of CNT internal fabrication (CNT-IF) and CNT surface modification (CNT-SM) membranes in terms of degradation efficiency (Figure 3a) and the flux profile (Figure 3b) for the filtration of humic acid. Under an applied voltage of −2.0 V, the CNT-IF membranes exhibited stable performance, retaining approximately 70% of their initial water flux over three cycles. This stability was attributed to the uniform distribution of CNTs within the membrane, which enhanced anti-fouling resistance. In contrast, the CNT-SM membranes experienced a decline in performance in the absence of voltage, retaining only approximately 50% of their initial flux. Additionally, the mechanisms were due to the fact that the even CNT distribution for CNT-IF (Figure 3c) promoted ROS generation for foulant degradation when compared with CNT-SM (Figure 3d) [48]. The defects in CNTs can act as catalytic active sites for pollutant degradation, which facilitates the donation of electrons [49]. CNTs can also be functionalized to enhance their electrocatalytic activity, such as CeO_2_@CNTs [50], SnO_2_-CNTs [51], and CNT/MoS_2_/Fe_3_O_4_ [52]. A CNTs/rGO cathode ECM was reported with good anti-fouling performance by self-assembly under vacuum filtration, ascribing to the electrostatic repulsion and electrocatalytic oxidation of humic acid by electro-generated H_2_O_2_ and the following •OH [53]. A CNTs/MXene ECM was prepared by loading CNTs and MXene onto a polytetrafluoroethylene (PTFE) membrane by layer-by-layer self-assembly, which restored flux to about 80% because of the degradation of foulants by •OH [18]. CNTs are also an efficient candidate to confine active sites, which have the benefits of exceptional catalytic activity and a nanoconfinement effect. For example, confining α-Fe_2_O_3_ into CNTs improved H_2_O_2_ selectivity via 2e^−^ ORR and generated singlet oxygen (^1^O_2_) for pollutant degradation [54].

Boron-doped diamond (BDD) is regarded as a nonactive anode for •OH production and presents metal-like conductivity, which presents unique advantages in the electrocatalytic degradation of pollutants and stability [55]. Nevertheless, the high cost, low electrocatalytic activity and porosity largely limit its application in ECMs [12]. Biochar and activated carbon are also widely applied in water treatment owing to their merits of porosity, low cost and rich functional groups, which can be fabricated from biomass (e.g., stalk, wood) and solid waste via carbonization [56]. Moreover, they are feasible for modification by activation using an acid and a base, or the introduction of carbon defects, while the insufficient available active sites and slow transfer rate retard oxidation efficiency. Instead, their porous nature makes them promising substrates for the loading of active sites (e.g., metal and metal oxide), which enables superior catalytic activity [56]. For instance, a Pd-loaded activated carbon anode ECM efficiently degraded pollutants by in situ-generated H* [57]. Metal oxide (CuO_x_, Fe_2_O_3_, and CoO) can be loaded on a coal-based carbon membrane by electrodeposition and oxidation methods [58]. Overall, carbonaceous materials played a dominant role in ECMs for fouling control and they usually required activation, introduced defects, and combined with other materials (e.g., metal oxide) to enhance the electrocatalytic activity toward pollutant degradation and inhibit competitive water splitting reactions (e.g., OER and HER).

### 2.2. Metal and Metal Oxide

Compared to carbonaceous materials, metal and metal oxide usually presented a higher OEP and an “nonactive” electrode, which favored ROS generation for water treatment. Therefore, metal and metal oxide were also widely investigated to construct ECMs for the degradation of foulants on a membrane. Noble metal (e.g., Au, Pd, Ag) is a kind of high-performance electrode material in electrochemical application. For example, Pd has exceptional H_2_ dissociation and atomic hydrogen (*H) adsorption ability, which was effective to apply in the *H-mediated electrochemical reduction of pollutants [59]. Zero valent Pd^0^ [60], Pd-PdO heterostructured nanotubes/a multilayer graphene anode [61] were prepared to enable efficient *H generation. To enhance the selectivity and Pd utilization efficiency, modification or doping with other metals is highly desirable to construct a high-performance ECM in general, such as Pd/NiAl-layered double hydroxide/carbon paper [62]. The modification of Pd with a rhodium nanoparticle by electrodeposition further enhances the adsorption of hydrogenation intermediates [63]. Bimetallic Pd and Fe nanoparticles were grown on CNTs to construct an ECM which can degrade a pollutant via atomic *H-mediated indirect reduction [64]. Nevertheless, the high cost and low abundance of the noble metals restricted their scalable application in ECMs for wastewater treatment.

To address the above issues, many attempts were made to reduce the loading of noble metals or replace them with non-noble metals. It is a promising method to reduce the loading of a noble metal without compromising the catalytic activity. Single-atom catalysts (SACs) emerged as a promising method to expose the active site and present high activity, where single metal atoms acted as the active sties, therefore realizing the almost complete utilization of active sites [65]. Therefore, the design noble metal-based SAC ECMs is promising to enable the above goal. However, until now, the literature has rarely reported this kind of ECM, likely owing to the fabrication factors and the future works needed to overcome this obstacle. In comparison, a non-noble metal ECM reduced the cost and provided promising choices for designing ECMs because of the abundant transition metal species [59]. Specifically, Ti_4_O_7_ with good electrical conductivity and chemical robustness received more attention, and acted as “nonactive” electrode with an OEP of 2.2–2.7 V and efficiently degraded pollutants [66]. Nevertheless, the low porosity and difficult scalability of Ti_4_O_7_ are still challenging. Other metal oxides (e.g., TiO_2_) generally have poor electrical conductivity, and they should be loaded or grown on a conductive substrate (e.g., carbon, metal) [67]. Benefiting from corrosion resistance and electrical conductivity, porous Ti mesh is commonly used as the conductive substrate for loading active materials (e.g., metal oxide, carbon or a heteroatom) to fabricate metal ECMs [68]. In addition, stainless steel, as a cost-effective material containing the intrinsically active elements (Fe, Ni, and Cr), is a promising candidate for the construction of metal-based ECMs [69]. Metal oxide (TiO_2_ and ZnO) ECMs can be prepared through atomic layer deposition on stainless steel [70]. Notably, Cr in stainless steel passivates during electrocatalysis, causing the deterioration of catalytic activity and electric conductivity [69]. Surface treatment or doping is essential to alleviate the electrochemical passivation of stainless steel. Recently, Fe-based electro-Fenton materials received intensive attention for ECM application and Fe catalyzed H_2_O_2_ to produce •OH for fouling control [71].

Although non-noble metals have attracted intensive attention in wastewater treatment, they have still presented inferior electrocatalytic activity compared to noble metals. Therefore, it is essential to develop an advanced strategy to enhance their performance. Single-atom catalysis is a powerful strategy to optimize atom efficiency and specific activity, and modulate selectivity and stability [72]. A single atomic Ti and titanium oxycarbide ECM with a 3D arrangement structure was prepared by loading into interconnected carbon nanofibers using a dual electrospinning-electrospraying-thermal post-treatment method [73]. The strong covalent binding and the localized ultrafast electron transfer between atomic catalysts with carbon ensured the excellent stability and electrocatalytic activity of the ECM [73]. A single atomic Ti and titanium oxycarbide ECM with fast electron transfer, a high specific surface and ECSA is effective in producing non-radical ^1^O_2_ through the electrocatalytic activation of dissolved O_2_ [73]. Notably, ^1^O_2_ presented a longer lifetime (3.5 μs) than •OH (<1 μs); moreover, it has wider pH adaptability, and superior resistance to environmental substrate interference and selectivity compared to common free radicals [74], making it a promising candidate for membrane fouling control. Nevertheless, the high loading of single atomic metals is still challenging for industrial application and they tend to aggregate when the loading amount is higher than 2% [72]. Moreover, the controllable synthesis of single atomic metal-based ECMs is another enormous task.

Considering the interdependent multi-step reaction and multiple intermediates (e.g., OOH*, O*, OH*) during electrocatalysis for the generation of ROS, a metal-based multi-site ECM has received increasing attention, which can inhibit the limitation of interdependence and provide abundant active sites for advanced catalysis [75]. For example, Ni-Cu bimetal was well dispersed on PES substrate using the co-reduction method, which exhibited high catalytic activity via the synergy of geometric and electronic properties [76]. The precise control of multiple active sites of metal still limited their application in ECM fabrication. In addition, the combination of metal with carbonaceous materials received increasing attention in ECM fabrication to resolve the issues of the conductivity and activity of pristine metal ECMs. For example, electrospinning followed by calcination were employed to fabricate SnO_2_@carbon fibers and Fe@carbon fibers with high conductivity and electron transfer efficiency, which were used as anodes and cathodes for the generation of ^1^O_2_ [77].

**Figure 4:**
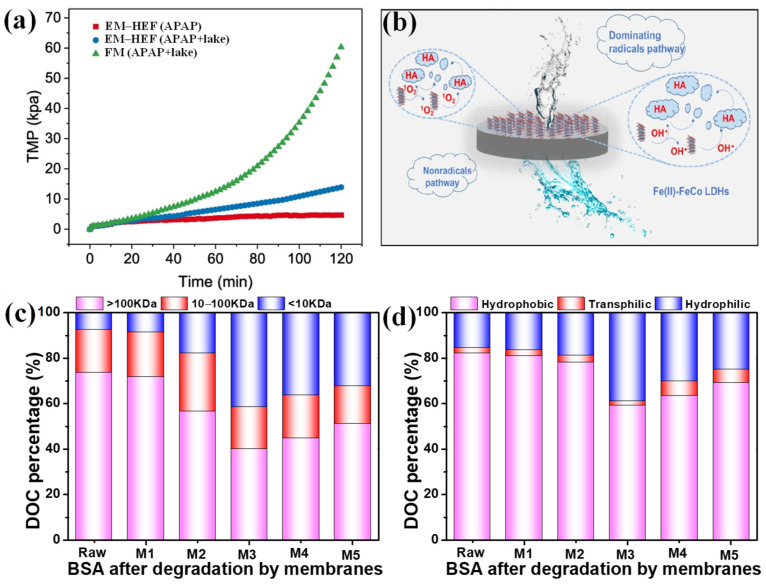
(**a**) The transmembrane pressure variation of an ACF/CC@FeOCl-Cu electron–Fenton membrane [78]; (**b**) Schematic diagram of the HA degradation mechanism in an electron–Fenton membrane [79]; Molecular weight distribution (**c**) and hydrophilicity/hydrophobicity (**d**) of bovine serum albumin (BSA) before and after degradation by the Fe and N co-doping biochar ECM [80]. APAP: Acetaminophen; EM-HEF: electro-controlled membrane coupling heterogeneous electro-Fenton; FM: flow-through membrane; HA: humic acid.

Fenton-based treatment (Fe^2+^ and H_2_O_2_) was widely used in wastewater treatment because of the high oxidation ability of •OH, while the harsh conditions (pH = 2–3), ferric sludge production and high chemical consumption were the bottleneck problems, which kept this from being a low-carbon water treatment technology. Electro-Fenton ECM technology is promising for breaking through the above bottlenecks through the in situ generation of H_2_O_2_ and the cycling the ferric ions under electricity, while low H_2_O_2_ selectivity and the sluggish Fe(II)/Fe(III) cycle still restrict the treatment efficiency. Modulation of the structure of Fe-based catalysts or Fenton-like catalysts can enhance the oxidation efficiency by optimizing the electronic structure and chemical microenvironment. An FeOCl-Cu nanosheet-loaded activated carbon fiber cathode ECM was fabricated to degrade natural organic matter (NOM) in lake water by •OH and repelled foulants through an electrostatic repulsive reaction, therefore enabling negligible transmembrane pressure enhancement (Figure 4a) [78]. Fe(II)-modulated FeCo-layered double hydroxide was effective in activating H_2_O_2_ into •OH and ^1^O_2_, which degraded humic acid in both the pores and surfaces of the membrane into smaller fragments, hence enabling superior fouling control (Figure 4b) [79]. Furthermore, the hydrophilic and rough interface of the ECM favored mass and charge exchange, and provided active sites, which is conductive to fouling control [79]. We reported an Fe and N co-doping biochar electro-Fenton ECM and confirmed that the presence of oxygen and nitrogen functional groups enhances the H_2_O_2_ selectivity via 2e^−^ ORR and boost the Fe(II)/Fe(III) cycle, hence achieving good self-cleaning performance, as evidenced by the almost 100% recovery of permeate flux [80]. The in situ generated •OH and •O_2_^−^ caused the partial mineralization (conversion into CO_2_ and H_2_O) of the organic foulants (bovine serum albumin) and reduced their molecular weight (Figure 4c) and hydrophobicity (Figure 4d), which weakened the interaction between foulants and the membrane and led to the easy detachment of foulants from the membrane by hydraulic cleaning. Recently, a metal-free electro-Fenton ECM has attracted more attention in membrane fouling control by introducing heteroatoms. The heteroatoms acted as active sites for H_2_O_2_ activation, which correspond with the foulants’ degradation and fouling control. A S and N self-doped carbon ECM was reported for the activation of H_2_O_2_ to ROS (•OH, •O_2_^−^, ^1^O_2_) in wide pH ranges (3–11) with an energy consumption of 12.5 W h g^−1^ [81]. The dopant effects of N and O heteroatoms on H_2_O_2_ generation were also confirmed by Wang et al. [82], who induced the electronic rearrangement and reduced the binding strength of *OOH. In addition, a rGO/poly(aminoanthraquinone) ECM can efficiently reduce flux decline by 63.5% for the UF membrane caused by BSA, ascribing to the electro-generation of H_2_O_2_ (8.84 mg/L) under an electric field of 1.0 V cm^−1^ [83].

To sum up, metal plays a crucial role in ECM materials with a diverse structure and rich active sites, while the possible leaching of heavy metal and the scalability of the ECM still remain the obstacles in their practical applications.

### 2.3. Conductive Polymer

**Figure 5:**
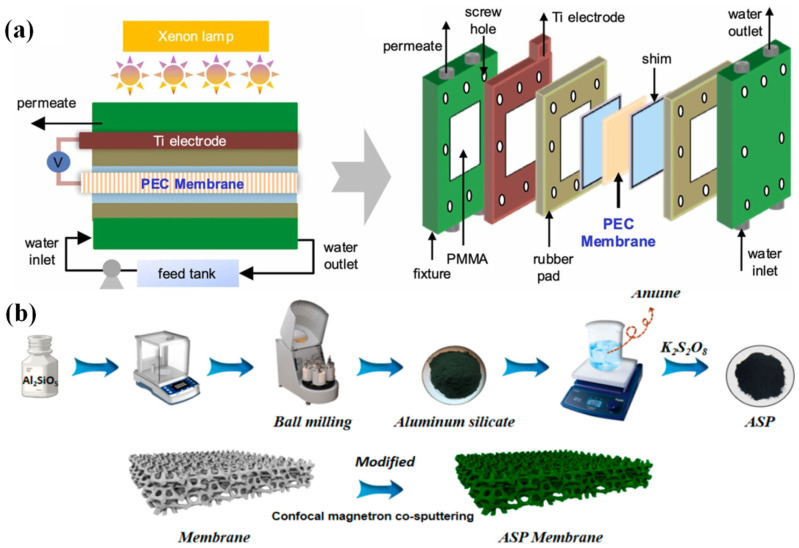
(**a**) Schematic diagram of cross-flow membrane reactor using TiO_2_/PANI/PVDF ECM [87]. (**b**) Synthesis route of PANI/aluminum silicate ceramic ECM [89].

A conductive polymer is another category of ECM material that has electrical conductivity and large-area fabrication. PANI and PPy are the two important conductive polymers for electrode membrane fabrication by polymerization using a monomer (aniline and pyrrole) [84]. However, the drawbacks of their insufficient oxidation ability, low active sites and porosity and poor mechanical stability still restricted their application in the electrocatalytic degradation of contaminants in wastewater [85]. Considering their strong comparability and facile synthesis, they usually combine with other materials for the construction of composite ECMs with higher conductivity, more accessible active sites and stronger ability. Immobilizing metal or doping heteroatoms on a conductive polymer can restrict the above drawbacks to enhance their porosity, activity and stability [84]. For example, polyaniline was coated on a coal-based carbon ECM by electrochemical polymerization deposition, which exhibited a lower water contact angle (30°), and higher water permeability (2564.67 L·m^−2^·h^−1^·bar^−1^) and OEP (1.65 V vs. SCE) compared to a pristine carbon ECM [86]. Similarly, a PPy-coated carbon-based ECM was also prepared by electro-polymerization deposition, which presented high water permeability (2073.1 L·m^−2^·h^−1^·bar^−1^) and the loading of PPy boosted the electron transfer efficiency for the production of •OH [23]. TiO_2_/PANI/PVDF ECM was also fabricated by phase inversion with an efficient self-cleaning ability during textile wastewater treatment with a cross-flow membrane reactor (Figure 5a) [87]. A PANI/carbon fiber can serve as a good substrate for the well dispersion of Pb, which increased the electrochemically active surface area (ECSA) by four-fold more than a conventional cathode ECM [88], suggesting that more exposed active sites are beneficial for the superior degradation of pollutants [16]. A PANI/aluminum silicate ceramic ECM was synthesized by polymerization and confocal magnetron co-sputtering (Figure 5b) [89]. A sheet-like aluminum silicate and rod-like PANI were observed from SEM images. Moreover, ROS (•OH, ^1^O_2_, •O_2_^−^) were detected during electrocatalytic degradation of the pollutant [89]. In addition to the above ECM materials, emerging materials have attracted increasing attention recently, which is promising for preventing the inherent drawbacks of conventional ECMs.

### 2.4. Emerging Materials

#### 2.4.1. MOFs

MOFs are composed of a metal cluster and organic ligand, which have become promising ECM materials because of their advantages of having high porosity, isolated active sites and a tunable structure [90]. MOFs can be synthesized by hydrothermal/solvothermal methods, mechanical methods, and microwave methods [91]. In situ growth of MOFs on a proper substrate is a common method for combining MOFs with membranes, which includes in situ growth at certain temperatures and the solvothermal method. For example, pure cellulose membranes were prepared using alkali/urea/thiourea/water as solvents, and ZIF-67 was loaded onto the membrane as a carrier. ZIF-67/cellulose hybrid membranes were prepared using an in situ growth method at room temperature, maintaining their good morphology, structure, and crystallization performance [92]. Similarly, CoFe-BTC nanoparticles were loaded on carbon fiber using a solvothermal method and the fabricated ECM had low ORR onset potential (0.054 V), which catalyzed H_2_O_2_ into •OH for the degradation of pollutants [93]. The ZIF-8/CNFs membrane demonstrated a certain level of adsorption capacity for pollutants, achieving adsorption equilibrium within a relatively short period of time [94]. The pseudo-first-order kinetic constants for pollutant degradation were determined to be 0.104 min^−1^ for ZIF-8 and 0.068 min^−1^ for ZIF-8/CNFs, respectively. This suggests that the incorporation of carbon nanofibers (CNFs) slightly diminished the catalytic efficiency of ZIF-8. Ultimately, a quenching test indicated that SO_4_^•−^ played a major role in the degradation of pollutants, rather than •OH, which was responsible for the degradation of pollutants [94].

**Figure 6:**
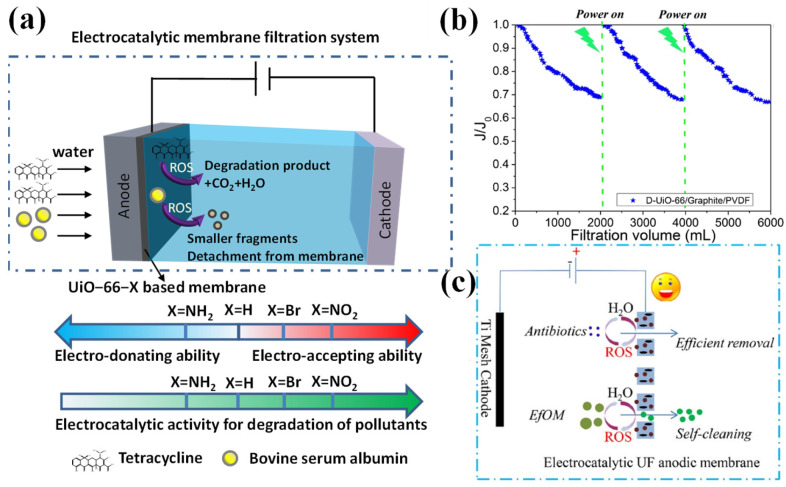
(**a**) Electronic effect on pollutants’ degradation by UiO-66-X (X = NH_2_, H, Br and NO_2_) electrocatalytic membrane [95]; (**b**) self-cleaning performance of UiO-66-D electrocatalytic membrane under current density of 0.01 mA/cm^2^; (**c**) schematic diagram of possible self-cleaning mechanism of UiO-66-D electrocatalytic membrane [96].

Modulating the electronic structure of MOFs is effective for optimizing the activity of MOF-based ECMs. We confirmed that the introduction of electro-drawing groups (-NO_2_ and -Br) into MOFs enhances the electrocatalytic activity of the ECM for the degradation of organic foulants by the electro-generation of •OH and •O_2_^−^, while the opposite phenomenon was observed for the electro-donating groups (-NH_2_) (Figure 6a) [95]. Meanwhile, considering that the active metal sites in MOFs were masked by binding with organic ligands, we utilized defects engineering to create exposed active sites and hierarchical pores in MOFs. This markedly enhanced the activity and degradation kinetics, and achieved complete flux recovery after self-cleaning (Figure 6b) with low energy consumption (0.457 W•h/L) owing to the degradation of foulants by electro-generated ROS (Figure 6c) [16,96]. Furthermore, to overcome the low electric conductivity of MOFs, we employed conductive MOF (Cu-HHTP) ECMs [97] and MOF/CNT ECMs [28] using the NIPS method, which enabled the antibiotic rejection and self-cleaning performance with almost 100% flux recovery after electrocatalytic treatment. Organic and inorganic foulants usually coexist in water matrices, causing severe fouling and forming a compact gel layer on the membrane surface. We further demonstrated that defective MOF-based ECMs can realize the efficient control of organic–inorganic combined fouling [9]. Flux was fully recovered by electricity and the fouling control mechanism can be ascribed to the destruction of calcium bridging action between the ECM and the biopolymer by in situ-generated ROS under electricity, followed by the release of calcium and degraded organic products from the membrane during hydraulic cleaning. Notably, the low stability of MOFs largely limited their application in electrocatalysis and their structure may transform during elctrocatalysis and acted as a precatalyst [29]. Recently, we systematically explored the role of MOFs’ structure and water matrices (mainly pollutant properties) on MOFs’ reconstruction during electrocatalytic water reclamation [98]. The results revealed defect-free that UiO-66 experienced the continuous collapse of the framework until being fully transformed to ZrOOH during the electrocatalytic degradation of pollutants in wastewater, which markedly reduced the catalytic activity and stability of the ECM. Unexpectedly, UiO-66 with a moderate concentration of missing-linker defects was quickly reconstructed into a ZrOOH@MOFs heterojunction with high activity and stability since the in situ-generated ZrOOH from exposed metal sites contained rich oxygen vacancy and prevented the further poisoning of MOFs [98]. In addition, the pollutant properties influenced the reconstruction degree of MOFs, depending on their physicochemical properties [98]. These observations guide the design of MOF ECM materials and their application in practical wastewater treatment and membrane fouling control.

Owing to the insufficient conductivity and stability, plenty of efforts were devoted to designing MOF derivations by pyrolysis or transformation, where MOFs were used as the precursor and template for the fabrication of a conductive ECM [89]. Although this strategy attracted more attention in the fabrication of ECMs, the loss of porosity of MOFs and the high energy consumption limit their practical application.

#### 2.4.2. MXene

MXene (M_n+1_X_n_T_x_), composed of transition metal carbides, nitrides, and carbonitrides, is a kind of two-dimensional material, which can be synthesized by bottom-up and top-down strategies [99]. It exhibits a hydrophilic nature, variable surface functionality, and good mechanical qualities, making it a promising ECM material. The combination of MXene with metal oxide and carbon-based materials can further enhance their catalytic performance, such as MXene/Ti_4_O_7_ [100] and Ti_3_C_2_T_x_-Cu [101]. Ti_3_C_2_T_x_/Ti ECM was constructed by electrophoretic deposition, which accelerated the production of •OH radicals [102]. A Pd-Cu/MXene ECM was also fabricated by vacuum filtration for nitrate reduction [103]. N-doping [104] and defect engineering [105] can further enhance the selectivity and reducibility of MXene for enhanced ROS generation. Sodium lignosulfonate carbon nanotubes/alkalized MXene composite membranes were developed to possess efficient anti-fouling and self-cleaning performance. Permeation flux was restored to about 80% by •OH after 10 min of electrocatalytic cleaning [18]. The triboelectric nanogenerator (TENG) technology can be used to reduce the energy consumption of ECM technology. The electricity can be self-generated for a CoFeMoOOH/MXene lamellar ECM by pulsed electricity cleaning, which activates peroxymonosulfate to ROS and thus efficiently alleviates the irreversible interlayer fouling with approximately 80% of flux recovery for oil–water separation [19]. Until now, the data in the literature about MXene-based ECM materials has been limited, which will attract more attention in membrane fouling control and pollutant removal for advanced water treatment.

## 3. Fabrication Methods of Electrocatalytic Membranes

Electrocatalytic membranes can be fabricated through many methods. Phase inversion is a facile and scalable method for the fabrication of ECMs by blending electrocatalytic materials into a polymer matrix. For example, a polyvinylidene fluoride (PVDF)-based ECM was constructed by introducing polydopamine-aminoquinone/reduced graphene oxide (PDAAQ/rGO) nanohybrids into a PVDF matrix [83]. Notably, electrocatalytic materials tend to aggregate in a polymer matrix and possible leaching may occur during long-term filtration, lowering the available active sites and deteriorating catalytic activity for foulant degradation and fouling control.

**Figure 7:**
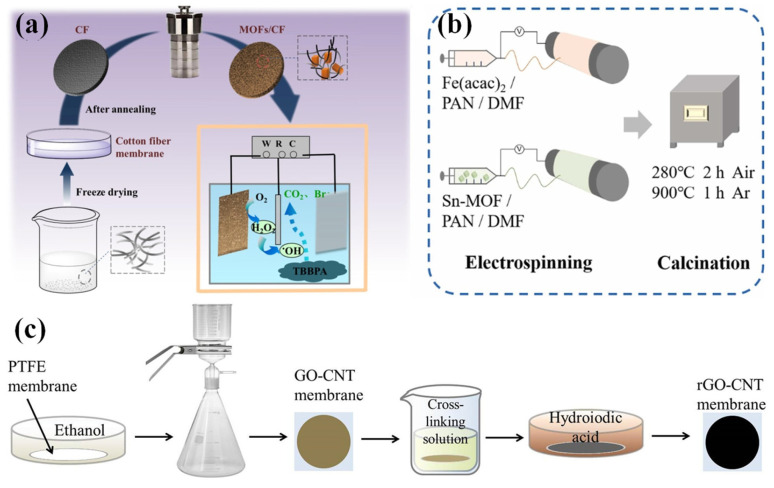
(**a**) The MOFs/CF electro-Fenton ECM created via hydrothermal synthesis [93]; (**b**) Fe@CF membranes created via the electrospinning-calcination method [77]; (**c**) the rGO-CNT membrane created via vacuum filtration [53].

The solvothermal method is characterized by placing the membrane and electrocatalytic materials in an autoclave to prepare an ECM at a certain temperature. For example, a CoFe-BTC/carbon fiber ECM is prepared by adding a carbon fiber into an autoclave containing Fe(NO_3_)_3_·9H_2_O, CoCl_2_·6H_2_O, trimesic acid (BTC) and HF at a certain temperature [106]. The CoFe-MOFs was grown in situ on a PAN membrane via a solvothermal method [107]. In addition, after hydrothermal treatment, Fe/Co bimetallic MOFs will grow in situ on the surface of carbon nanofibers (CNFs) [108]. MOFs were immobilized on carbon fiber (CF) substrates by in situ hydrothermal growth [93] (Figure 7a). The directional and uniform growth of electrocatalytic materials on a substrate is important to designing a high-performance ECM, which is meaningful to devote more efforts to in future studies.

In addition, electrospinning is also a powerful tool to construct a fiber-based ECM. For example, iron-incorporated carbon nanofiber (Fe@CF) membranes were constructed by an electrospinning-calcination method [77] (Figure 7b). In addition, vacuum-assisted filtration is a more suitable method for loading electrocatalytic materials (especially for two-dimensional materials, e.g., MOF nanosheets, GO and MXene) onto CNF membranes than an in situ method. These techniques enable precise control over membrane composition, structure, and catalytic functionality. For example, reduced graphene oxide/carbon nanotube (rGO/CNT)-layered architectures were constructed by vacuum-assisted filtration [53] (Figure 7c). The preparation of Co/Fe MOFs/CNF membranes involves the fabrication of Co/Fe bi MOFs and the filtration of MOFs through carbon nanofibers, and the removal of solvent by freeze drying [109]. Similarly, MIL-100 [110] and ZIF-8/CNFs [94] were also incorporated into the polymer membrane by the vacuum filtration method and the unloaded materials were rinsed off with ethanol several times. Although vacuum-assisted filtration has been widely used in the lab-scale fabrication of ECMs, its industrial and scalable fabrication have still been challenging and the low interaction between active materials and substrates caused the poor stability of the ECMs. Therefore, additional cross-linking or combination with other methods (e.g., interfacial polymerization) is necessary to address the above limits [111]. Each method has advantages and disadvantages for the fabrication of ECMs and more emerging methods hold great potential to fabricate ECMs in order to meet the requirements of targeted pollutant removal and sustainable fouling control with high stability and low energy consumption.

## 4. Electrocatalytic Self-Cleaning Performance and Mechanism

### 4.1. Effect of Foulant and Wastewater Types

Notably, there are many types of wastewater, such as municipal wastewater, industrial wastewater (textile dyeing wastewater, tannery wastewater) and aquaculture wastewater [7]. Moreover, plenty of foulants with diverse structures and properties exist in different water matrices, including inorganic foulants, organic foulants, colloidal foulants, microbial foulants and combined foulants, causing different membrane fouling types and behaviors [2,8]. This brings great challenges to membrane fouling control and it is important to clarify the suitability of each electrocatalytic membrane type for the treatment of these wastewaters containing different foulants types.

Most studies employed organic fouling to evaluate the self-cleaning performance of ECMs. For example, humic acid fouling of an FeOCl@NCNT/ceramic membrane can be alleviated by electro-Fenton catalysis [14]. Nano-zeolite/carbon nanostructure ceramics enabled efficient bio-fouling control performance by applying periodic electricity [112]. Notably, many kinds of foulants have normally coexisted in wastewater and caused the more serious combined fouling. For example, calcium ions bound with polysaccharide, which formed a compact gel layer on the membrane. This required more energy consumption to enable the complete recovery of the flux of a MOF-based ECM caused by Ca^2+^-alginate combined foulants compared to the individual alginate fouling [9]. We also confirmed that MOF-based ECMs utilized the efficient self-cleaning performance without obvious flux loss during the long-term treatment of municipal wastewater secondary effluent over 30 days [16]. Therefore, the ECM has potential to control the fouling caused by different kinds of foulants in wastewater. The optimization of ECM materials and operating conditions will further improve the self-cleaning performance.

### 4.2. Self-Cleaning Mechanism

An ECM can act as anode or cathode in an electrocatalytic membrane system, where different reactions have occurred. The self-cleaning mechanism is mainly caused by the degradation of foulants on an ECM in a direct or indirect pathway. The direct pathway indicated the direct electron transfer between foulants and the ECM. Direct electrooxidation of foulants may occur through electrons transferring directly from foulants to the anodic ECM, therefore selectively degrading foulants that readily donate electrons [12]; moreover, foulants may be directly reduced by a cathodic ECM. Generally, direct and indirect pathways both contributed to foulants’ degradation. For example, Gayen et al. developed a Bi-doped SnO_2_-Ti_n_O_2n–1_ anodic ECM for the degradation of pollutants via the synergy of direct electron transfer and direct electrooxidation by •OH [113].

Compared with the indirect pathway, the indirect degradation of foulants was more widely studied to alleviate the fouling of ECMs. Reactive oxygen species (e.g., •OH, •O_2_^−^, ^1^O_2_, SO_4_^•−^) and reactive chloride species (e.g., Cl•, ClO•) with high oxidation potential were observed to be produced on anodic ECMs under electricity, which were responsible for foulant degradation and membrane fouling control [12,13,66]. A TiO_2_-based anodic ECM with moderate oxygen vacancies promoted ROS generation because of the moderate adsorption of *OOH and *OH [67]. However, when the oxygen vacancies’ concentration was excessive, the ROS production was inhibited owing to the difficult desorption of *OOH and *OH from the active sites of the ECM [67]. A cathodic ECM can generate H_2_O_2_ through 2e^−^ ORR, which can enable the in situ cleaning of the membrane [36]. In addition, the electrogenerated H_2_O_2_ can be activated by Fe^2+^ on a cathodic ECM to produce •OH, while Fe^3+^ was reduced to Fe^2+^ by accepting an electron on the cathodic ECM. The above cathodic ECM was called an electro-Fenton membrane. The high H_2_O_2_ selectivity and fast Fe^2+^/Fe^3+^ cycle were the important factors influencing the pollutant degradation efficiency and self-cleaning performance of the ECM [54,71,78]. Notably, organic foulants on the membrane are difficult fully mineralize by the direct or indirect degradation pathway. Normally, the self-cleaning mechanism is owed to the partial mineralization and the degradation of foulants into smaller fragments with low adhesion with the membrane by direct electron transfer or indirect degradation by ROS, thereby removing from the membrane by hydraulic cleaning [79,80,96,97]. Moreover, the membrane materials influenced the direct and indirect pathway during the electrocatalytic degradation of foulants, therefore governing the membrane self-cleaning mechanism.

## 5. Challenges and Future Prospects

We provided a summary table for the membrane materials and their performance in Table 2. Although different kinds of ECM materials were fabricated and developed in past decades, the low cost and scalability of fabrication were still not satisfactory and many studies usually overlooked these issues. Moreover, most of studies were at a lab scale and the pilot plant and large-scale applications were still rare in practical wastewater treatment and membrane fouling control. Long-term filtration at a full scale should be conducted in the future in order to resolve the above obstacles. Nanomaterials played a crucial role in ECM fabrication, but their aggregation and leaching issues should be addressed. Moreover, the interaction between the active materials and the substrate should be emphasized. In many studies, the membrane only acts as the substrate of catalysts for the degradation of pollutants, while their separation function is underestimated. The precise control of the ECM structure and chemical environment is an important trend to optimize the activity and stability of the ECM with efficient water treatment and a high self-cleaning ability. The ECM materials genome will be established using machine learning.

The development of ECM-based water treatment facilities was another huge challenge in industrial application. Flow-through operating mode has received more intensive attention in the ECM process than flow-by mode owing to its fast mass transfer through convection, which increases the turbulence intensity to 200% and the mass transfer coefficient [32]. A tubular flow-through ECM reactor was designed for pollutant degradation and the CA (cathode to anode) flow direction overwhelmed with a 40–50% degradation efficiency compared to AC (anode to cathode) owing to the different pH distributions [114]. The combination of electricity and the membrane module (e.g., roll to roll, hollow fiber) is challenging. The electroactive flat membrane can be rolled into multiple layers as the anode and the active layer faces the outside. Then the Ti metal sheet is rolled into a cylinder for securing both sides of the rolled membrane and counter electrode in order to transfer the current to the membrane. Meanwhile, the wire mesh was bent into a cylindrical shape as the counter electrode of the electrocatalytic membrane module. The counter electrode and the membrane are connected with a power supply with an adjustable distance. For the self-cleaning process, external electricity was applied and the self-cleaning performance can be optimized by adjusting the electricity condition and the flow rate of backwashing [28]. Nevertheless, the current ECM facilities still present an obvious gap with the cross-flow filtration in the membrane module in large-scale applications and membrane fouling is another important drawback for conventional flow-through mode, which facilitates the accumulation of foulants on the membrane. In addition, although the chemical consumption is low for ECM technology, the energy consumption is enhanced, thus there was a balance between the operating cost and chemical cost. The electricity can be produced during membrane filtration for some ECM materials via the piezoelectric effect and the triboelectric nanogenerator [115], which holds great potential in designing a self-cleaning ECM with low energy consumption.

In addition, most studies investigated the self-cleaning performance of ECMs using model and single foulants. The structure and properties of the above model foulants were different from the foulants in real wastewater, therefore the ECM fouling control performance from the aspects of model foulants may fail to guide practical application or even cause a misunderstanding of fouling [7]. In other words, the good self-cleaning of an ECM for the filtration of model foulants in simulated wastewater does not mean the same performance in practical wastewater. For example, bovine serum albumin (BSA) and sodium alginate (SA) are common model foulants of protein and polysaccharides, respectively [33], which have different molecular weights and physicochemical properties with the organic foulants in wastewater or surface water. Additionally, different kinds of foulants (e.g., inorganic, organic, biological and colloidal ones) coexist in real wastewater, which causes more severe combined fouling. Thus, the self-cleaning performance of ECMs in different water matrices still remains unclear. In addition, the in-depth research into self-cleaning and the membrane fouling control mechanism should be conducted from the electron and atomic level with the help of advanced analyses and theoretical calculation.

**Table 2:** The electrocatalytic membrane materials and their fouling control performance.

ECM Materials	Water Composition	Operating Conditions	Fouling Control Performance	Reference
D-UiO-66/PVDF	100 mg/L BSA; 100 mg/L SA; 0.5 mM Ca^2+^	Current density = 0.01 mA/cm^2^; Energy consumption = 0.46 W·h/L;	flux restored nearly 100 % after three fouling self-cleanings	[9]
CNT/MXene	20 mg/L methylene blue	Voltage = 4 V; Flux = 37.51 L·m^−2^·h^−1^·bar^−1^	membrane’s permeation flux recovered to about 80% after 10 min	[18]
Fe-g-C_3_N_4_/ABC-600/Graphite/PVDF	100 mg/L BSA; pH = 7.0	Flux = 421.3 L·m^−2^·h^−1^·bar^−1^; Energy consumption = 0.199 W·h/L; Residence time = 0.749 min; Excellent self-cleaning property	flux loss was negligible	[80]
Ti-MOFs-1	Simulated municipal wastewater; yeast extract (22 mg/L), peptone (32 mg/L), urea (6 mg/L)	Current density = 2.0 mA·cm^−2^; Flux = 618.6 L·m^−2^·h^−1^·bar^−1^; EC = 0.533 W·h/m^3^; Residence time = 50.2 s; Excellent self-cleaning property	Normalized flux decreased to 90% after 48 h operation	[96]
Reconstructed UiO-66	Surface water, pH = 7.34	Voltage = 1.5 V, flux = 1223 L·m^−2^·h^−1^·bar^−1^, continuous operating mode	no obvious reduction in flux	[98]
Graphene modified membrane	Pollutant: 20 mg/L alginate; pH = 7.0 0.05 mM Na_2_SO_4_	Voltage = −2.5 V; Residence time = 7.5 min;	Normalized flux decreased to 76% after 10 h of operation	[116]
FeNi-layered double hydroxide/carbon nanotube-based membrane	Humic acid; pH = 6.8 100 mM Na_2_SO_4_	Current density = 2 mA cm^−2^; Flux = 225 L·m^−2^·h^−1^·bar^−1^; Energy consumption = 0.331 W·h/L;	Normalized flux decreased to 70% after 1 h operation	[117]
PANI-CNT	100 mg/L BSA	Voltage = 3 V; Flux = 40 L·m^−2^·h^−1^	81% flux recovery	[118]

## 6. Conclusions

Important progress in self-cleaning ECM technology has been made in water treatment and ECM materials with different structures and compositions were developed in recent decades. We provide information on the features of recent advances in ECM materials, and their fabrication and application for sustainable fouling control. Carbonaceous materials, metal and metal oxide predominate the ECM materials, which have inherent drawbacks and merits, thus the combination of different materials is widely used to enhance the activity and stability of ECMs for pollutant degradation. Recently, emerging ECM materials were also developed with high performance and there will be further investigations of these in the future. The operating modes and water matrices also influenced the performance of the ECMs. Although ECM technology has gained importance in water treatment and fouling control, there were still some challenges in the aspects of cost, scalability, membrane module, stability and the self-cleaning mechanism. We believe that ECM materials will be optimized and ECM technology will play a more important role in many application scenarios.

## Data Availability

We have provided all the data supporting the reported results.
